# Respiratory Syncytial Virus Prevention through Monoclonal Antibodies: A Cross-Sectional Study on Knowledge, Attitudes, and Practices of Italian Pediatricians

**DOI:** 10.3390/pediatric15010013

**Published:** 2023-02-20

**Authors:** Matteo Riccò, Silvia Corrado, Milena Pia Cerviere, Silvia Ranzieri, Federico Marchesi

**Affiliations:** 1 AUSL–IRCCS di Reggio Emilia, Servizio di Prevenzione e Sicurezza Negli Ambienti di Lavoro (SPSAL), Local Health Unit of Reggio Emilia, 42122 Reggio Emilia, Italy; 2 Department of Medicine DAME–Division of Pediatrics, University of Udine, 33100 Udine, Italy; 3 UOC of Endocrine and Metabolic Surgery, Fondazione Policlinico Universitario Agostino Gemelli IRCCS, 00168 Rome, Italy; 4 Department of Medicine and Surgery, University of Parma, 43126 Parma, Italy

**Keywords:** RSV, RSV all infants, RSV epidemiology, RSV pediatric burden, RSV prevention, RSV vaccines, monoclonal antibodies, respiratory syncytial virus

## Abstract

Respiratory Syncytial Virus (RSV) is a leading cause of morbidity and hospitalization in all infants. Many RSV vaccines and monoclonal antibodies (mAb) are currently under development to protect all infants, but to date preventive options are available only for preterms. In this study, we assessed the knowledge, attitudes, and practices towards RSV and the preventive use of mAb in a sample of Italian Pediatricians. An internet survey was administered through an internet discussion group, with a response rate of 4.4% over the potential respondents (No. 389 out of 8842, mean age 40.1 ± 9.1 years). The association of individual factors, knowledge, and risk perception status with the attitude towards mAb was initially inquired by means of a chi squared test, and all variables associated with mAb with *p* < 0.05 were included in a multivariable model calculating correspondent adjusted Odds Ratio (aOR) with 95% confidence intervals (95%CI). Of the participants, 41.9% had managed RSV cases in the previous 5 years, 34.4% had diagnosed RSV cases, and 32.6% required a subsequent hospitalization. However, only 14.4% had previously required mAb as immunoprophylaxis for RSV. Knowledge status was substantially inappropriate (actual estimate 54.0% ± 14.2, potential range 0–100), while the majority of participants acknowledged RSV as a substantial health threat for all infants (84.8%). In multivariable analysis, all these factors were characterized as positive effectors for having prescribed mAb (aOR 6.560, 95%CI 2.904–14.822 for higher knowledge score; aOR 6.579, 95%CI 2.919–14.827 for having a hospital background, and a OR 13.440, 95%CI 3.989; 45.287 for living in Italian Major Islands). In other words, reporting less knowledge gaps, having worked in settings with a higher risk of interaction with more severe cases, and being from Italian Major Islands, were identified as positive effectors for a higher reliance on mAb. However, the significant extent of knowledge gaps highlights the importance of appropriate medical education on RSV, its potential health consequences, and the investigational preventive interventions.

## 1. Introduction

Human Respiratory Syncytial Virus (RSV) is a common and contagious pathogen that belongs to the genus orthopneumovirus (family Pneumoviridae) [1,2,3]. Globally, RSV represents a leading cause of lower respiratory tract infections (LRTI) in infants aged 2 years or less [1,2,3], most of them being otherwise healthy children [4,5], with a well-defined seasonal trend [2,6]. Even though a limited share of the infected infants eventually develop severe features (i.e., bronchiolitis and pneumonia), RSV infections are usually characterized by high hospitalization rates [7,8,9,10]. Albeit often acknowledged as nothing more than a pediatric pathogen [2,6,11,12,13,14,15], RSV also causes severe clinical features in older individuals, particularly in adults 65 years and older, as well as among adults with chronic heart or lung diseases, and/or a weakened immune system, representing a main cause of morbidity and mortality, particularly among institutionalized subjects [16,17]. 

Despite intensive research efforts, the appropriate management of RSV illnesses in all infants remains quite complicated [18]. On the one hand, no etiological therapy has been made available [15,19]. On the other hand [20], more than 60 years of intensive research has failed in providing safe and effective vaccines [20] that still remain commercially unavailable [2,11,16,21,22]. For nearly two decades, the only preventive option available has been therefore based on the passive immunization through the humanized monoclonal antibody (mAb) palivizumab (SYNAGIS^®^; USA approval 1998, EU approval 1999) [4,23,24,25,26,27]. Palivizumab specifically targets the domain A in the fusion (F) protein of RSV, inhibiting the viral attachment and the initial stages of infection [23]. The drug is delivered through monthly injections of a weight-dependent dose (i.e., 15 mg/kg) during the months characterized by a high circulation of the pathogen (“RSV season”), in up to five consecutive doses [4,5,24,25,26,28]. Despite its proven efficacy, this is a relatively expensive medication, as the cost for a 100-mg vial usually ranges from 904$ to 1866$ [4,5,24,25,26,28]. Moreover, guideline indications have become increasingly restrictive, and a palivizumab license is currently limited to some high-risk groups [10,16,27,29,30,31], including: (a) infants born at ≤ 35 weeks of Gestational Age (wGA); (b) children < 2 years of age affected by chronic lung disease of prematurity (CLD); and (c) children < 2 years of age affected by hemodynamically significant congenital heart disease (CHD) [32,33,34]. Nevertheless, not only have recommendations changed over the years, but stronger limitations have been issued depending on countries’ public health policies [25,26,28]. More recently, an extended half-life recombinant mAb, i.e., nirsevimab (MEDI8897; commercial name: Beyfortus^®^), has been shown as quite effective in reducing the risk for medically attended RSV infections (i.e., 74.5%; 95% Confidence Interval [95%CI] 49.6 to 87.1) [35], and hospitalizations (i.e., 78%) [36], being approved in the EU for the prevention of RSV-associated LRTI in newborns and infants from birth during their first RSV season [35,36,37]. Similarly to palivizumab, nirsevimab targets the F-protein [35,36,37,38,39] on a quite different domain (i.e., domain ø vs. A), and exhibiting a significantly higher activity (>50-fold) [5,11,38,39]. Because of its extended half-life, the recommended dose of nirsevimab is a single intramuscular injection of 50 mg for infants with body weight <5 kg, and a single intramuscular injection of 100 mg for infants with body weight ≥5 kg [5,11,38,40].

During the early stages of the ongoing SARS-CoV-2 pandemic, the extensive implementation of lockdown and physical distancing (collectively: non-pharmacological interventions or NPI) have shown a substantial and somehow unexpected efficacy in limiting the circulation of all respiratory pathogens, including RSV [41,42,43,44,45,46]. [41,42,43,44,45] Nevertheless, the lifting or even the removal of NPI during the second half of 2020 has been coupled with the rapid resurgence of RSV infections, followed by an unprecedented peak of new infections and hospital admission rates during the RSV season 2020–2021 [47,48,49]. As a consequence, RSV resurgence has led to an unprecedented workload for all pediatricians, and particularly for those involved in the hospital management of RSV-related LRTI cases [42,49,50]. Still, little is known about their actual understanding of the prophylactic options guaranteed by mAb [35,36,37,38]. Moreover, while previous studies have specifically inquired medical and alleged professionals about RSV infection and tentative vaccines [51,52,53], their actual experiences with mAb in the prevention of RSV largely remains unascertained. As the physicians’ knowledge, attitudes, and practices (collectively, KAP) are critical in modelling the acceptance of any intervention [54], we specifically inquired a sample of Italian pediatricians on their understanding of RSV disease, their practices regarding the management of RSV disease, and their previous use of mAb as prophylactic options.

## 2. Materials and Methods

### 2.1. Study Design 

As a follow-up of a previous cross-sectional study [51], we designed a questionnaire-based survey according to the STROBE guidelines (Strengthening the reporting of observational studies in epidemiology; see STROBE checklist as Supplementary Material S1) [55]. The present survey was shared as a web-based questionnaire between 7 April 2022 and 22 April 2022. It involved Italian medical professionals participating in three closed Facebook discussion groups belonging to the Facebook Community “Memedical”, a mutual help community for Italian medical professional that was founded during the SARS-CoV-2 pandemic [56]. In total, by 7 April 2022, the groups had a total of 8842 unique members, encompassing medical professionals from various specialties and subspecialties, as well as medical settings (primary care, hospitals, etc.). In order to be admitted within the group, all professionals must share with the discussion group leaders their registration number to the competent local medical board, allowing the moderators to check their professional status through the specifically designed web-based App of the Italian Federation of Medical Boards (FNOMCEO; https://portale.fnomceo.it/cerca-prof/ (accessed on 24 September 2022)).

Before the inception of the survey, a preventive authorization for posting the study invitation within the group was requested by the chief researcher (MR). The invitation post included a summary of the aims of the survey and a direct link to the questionnaire (Google Forms; Google LLC; Menlo Park, CA, USA). By accessing the first page of the questionnaire, the participant received the full informed consent, outlining the purpose, the risks, and the potential benefits of the study (Supplementary Material S2). No personal data such as name, IP address, or email address were requested, saved or tracked. No monetary or other compensations were offered to the participants.

### 2.2. Inclusion Criteria

Participation was offered to all of the participants from the discussion group, but only respondents expressly opting for participation were allowed to further proceed to the survey. To be included in the sample, the respondents were supposed to be (1) qualified medical professionals working in pediatric settings and (2) living and working in Italy by the time of the survey. Inclusion criteria were assessed by a dichotomous question (yes vs. no) that was self-reported and not externally validated. If the participant did not fulfill the aforementioned inclusion criteria, the questionnaire closed down without any further question.

### 2.3. Sample Size

As no KAP studies on the use of mAb in RSV cases have been previously performed on Italian pediatricians, minimum sample size (N) was calculated by cautiously assuming: an expected prevalence of 0.5 for having previously cared for RSV cases, a Type I error of 5% (0.05), and a power of 95%. As a consequence, the sample size was estimated as follows:

N = 1.96^2^ × 0.5 × (1 − 0.5)/0.05^2^ = 3.8416 × 0.8 × 0.2/0.0025 = 384

### 2.4. Questionnaire

The questionnaire was validated in a preliminary study on the acceptance of a tentative RSV vaccine, and its characteristics have been published elsewhere [51]. Briefly, it was formulated through an extensive review of the available literature on RSV [1,2,10,11,12,57,58,59,60,61,62,63,64], and included the following sections (Supplementary Table S1):

1. Characteristics of the participants: age, sex, seniority, Italian region where the professional mainly worked and lived.
2. General Knowledge Test. A series of 25 statements were shown to the study participants (i.e., 19 true-false; 6 multiple-choice). A cumulative score (General Knowledge Score; GKS) was calculated by adding +1 for every correct answer, with a potential range 0 to 25. A similarly designed knowledge test was previously applied for KAP studies in healthcare settings and effectively adapted to a broad array of medical settings [54,65,66,67,68].
3. Risk perception. According to the original definition of Yates, the perceived risk may be acknowledged as the function of the perceived probability of an event (F) and its expected consequences (C) [69]. Participants were therefore asked to rate the perceived severity (C) and the perceived frequency (F) of RSV infections through a fully labeled 5-points Likert scale (range: from “not significant”, 1, to “very significant”, 5). Distinctive estimates were calculated for infants (age 0 to 8 years), adults (age 18 to 64 years), and elderly (age 65 and more). Three Risk Perception Scores (RPS) were therefore calculated as the mathematical product of C and F (i.e., RPS = C x F, potential range 1 to 25).
4. Attitudes towards mAb. Participants were initially asked to self-rate their attitude towards RSV mAb as a prophylactic option. Respondents were then asked whether they acknowledged mAb as a valuable option for preventing RSV natural infection, and for avoiding severe infections including LRTI. All of the aforementioned items were rated through a 5-points fully labeled Likert scale that ranged from “totally disagree” (1) to “totally agree” (5). Attitudes were then dichotomized in “somewhat agreeing” (i.e., agree to totally agree) vs. “somewhat disagreeing” (i.e., totally disagree to neutral).
5. Practices. Participants were eventually asked about their interactions with RSV in the previous 5 years, and more precisely if they: (a) managed any RSV case in their daily practice; (b) diagnosed at least one case of RSV infection; (c) required any hospitalization for LRTI cases associated with RSV cases infections; or (d) required any shot of mAb for RSV immunoprophylaxis. All the aforementioned iterations were assessed as dichotomous items (i.e., ever vs. never).

All aforementioned items were self-reported, and not externally validated. The Authors’ translation is available as Supplementary Table S1.

### 2.5. Ethical Considerations

Through the informed consent, participants were preventively briefed about the aims and design of this survey, and only individuals acknowledging their agreement participated in the study. Retrieved data were handled anonymously and confidentially by means of the anonymous, observational design. As participants cannot be individually identified through the presented material and retrieved demographic data, and the present survey did not include clinical data about participants, the present study reasonably caused no harm or stigma to the participants, and a preliminary evaluation by an Ethical Committee was not forcibly required according to the Italian law (Italian Official Journal. 76, dated 31 March 2008).

### 2.6. Data Analysis

First of all, sum scores (i.e., GSK, RPS for infants, adults, and elderly) were normalized to percent values, and then dichotomized in “high” vs. “low” estimates by correspondent median values. Descriptive analysis was then performed by reporting categorical variables as percent values, while continuous variables were reported as average ± standard deviation (SD).

Univariate analysis of continuous variables required the preventive assessment of their distribution through the D’Agostino and Pearson omnibus normality test. A *p* value equals to 0.10 was assumed as cut-off value for Gaussian (“normal”) vs. non-Gaussian (i.e., “non-normal”) distribution. Continuous variables scoring a D’Agostino and Pearson *p* value ≥ 0.10 were compared using the Student’s t test or ANOVA, and their correlation was assessed by means of Pearson’s correlation coefficient. Conversely, variables not passing the normality test (*p* < 0.10) were analyzed by means of non-parametric tests (i.e., Mann–Whitney or Kruskal–Wallis test for multiple independent samples; and Spearman’s rank correlation coefficient).

Distribution of categorical variables was initially analyzed through chi-squared test in respect of previous interaction with RSV cases (any vs. never). Further analyses were performed only on cases that had previously managed any RSV, by focusing on their previous use of mAb (i.e., ever vs. never). Internal consistency of the knowledge test also estimated through calculation of Cronbach’s alpha, a statistic calculated from the pairwise correlations between items. In general, a score ≥ 0.7 is considered acceptable.

All categorical variables that at univariate analysis were associated with the aforementioned statuses with a *p* value < 0.10 were included as explanatory variables in two distinctive stepwise binary logistic regression analysis models: (a) model 1: outcome variable, represented by having previously managed any RSV case; (b) model 2: having previously employed RSV mAb as preventive therapy. 

Corresponding multivariable adjusted odds ratios (aOR), and their respective 95% confidence intervals (95%CI) were calculated accordingly. All statistical analyses were performed by means of IBM SPSS Statistics 26.0 for Macintosh (IBM Corp. Armonk, NY, USA), while plots were calculated in R (version 4.0.3) [70], and RStudio (version 1.4.1717; RStudio, PBC; Boston, MA, USA) software (packages *ggpubr* and *ggplot2*).

## 3. Results

### 3.1. Descriptive Analysis: General Characteristics of the Sample

**Figure 1:**
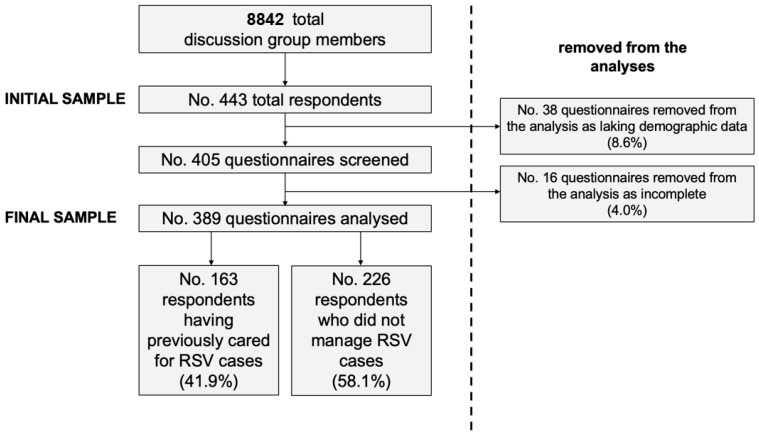
Flow chart of the selection of study participants.

From a potentially eligible population of 8842 medical professionals, a total of 443 pediatricians (5.0% of the potentially eligible population) signed the informed agreement granting their participation into this study (Figure 1). Of them, 389 (87.8% of all respondents, and 4.4% of the original population) reportedly worked as pediatricians either in hospital settings or not, fulfilling inclusion criteria. Of them, 163 (41.9%) had cared for RSV cases in the previous 5 years, while the majority had not (No. = 226, 58.1%).

The majority of pediatricians included in the final sample were females (61.2%), from Northern Italy (i.e., North-Western Italy, 17.7%; North-Eastern Italy 32.6%), with a mean age of 40.1 years ± 9.1 years, and a total seniority (including the years of post-degree qualification) of 13.9 ± 9.0 years (Table 1).

### 3.2. General Knowledge Test

**Figure 2:**
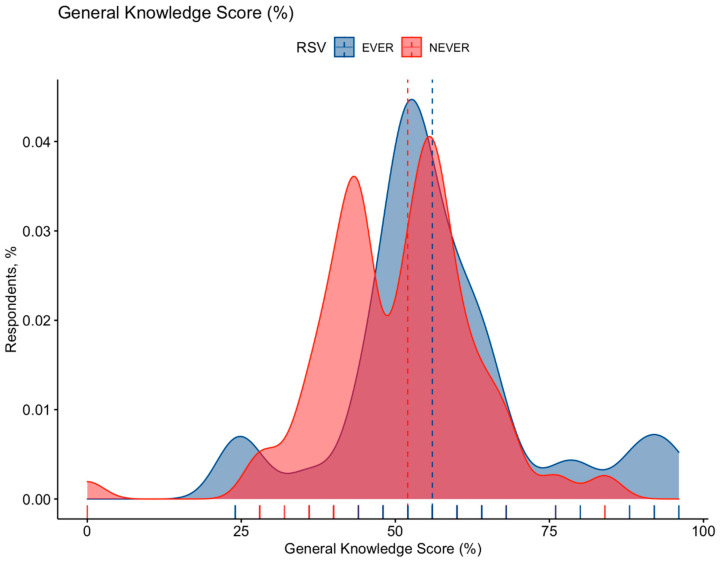
Density plot for General Knowledge Score (GKS) in 389 Italian Pediatricians participating into the survey, broken down by having or not having cared for Respiratory Syncytial Virus (RSV) cases in the previous 5 years. Cumulative score was substantially skewed for GKS (D’Agostino–Pearson’s normality test: *p* = 0.084). GKS was substantially greater among participants that had previously cared for RSV cases (56.6% ± 15.5) than among those having not (50.4% ± 12.6, Mann–Whitney test *p* value < 0.001).

A detailed report of the knowledge test is shown in Supplementary Table S2. Briefly, GKS was quite unsatisfying (54.0% ± 14.2; median 52.0%), and its distribution was somewhat skewed (D’Agostino–Pearson normality test, *p* = 0.084) (Figure 2). However, Cronbach’s alpha was estimated in 0.701, suggesting an acceptable internal consistence of the questionnaire.

In fact, while participants were mostly aware of the seasonal trend (61.4%, with 23.1% reporting on an RSV season lasting from October to February), and the current epidemiological transition of RSV during the SARS-CoV-2 pandemic (i.e., 64.8% properly acknowledged the global decrease in incidence rates, while 73.3% correctly reported on the RSV epidemic during the winter season 2021), the actual features of RSV infections were inconsistently reported. On the one hand, the majority of participants exhibited a proper understanding of both short- and long-term complications of RSV, not only in terms of their higher occurrence compared with seasonal influenza infections (73.0% of correct answers), but also when focusing on the reported neurological complications (70.4% of correct answers), and on the potential role of RSV infections in the of etiology of asthma (79.4%). On the other hand, the large majority of participants did not associate RSV-related deaths with older age groups (22.4% of correct answers), and they were mostly unable to correctly recall the actual rate of RSV-related hospitalizations (40.1%), particularly in the first year of age (0.5 per 100; 16.7%). Other common mistakes were represented by not acknowledging that the majority of RSV-associated hospitalizations do not occur among pre-term infants (25.4%), not being restricted to children with chronic respiratory disorders and cardiac malformations (40.1%), and that a high share (i.e., 60%) of pediatric LRTI is actually associated with RSV infections (42.2% of correct answers). Nevertheless, the total RSV-associated deaths in infants < 1 year of age were largely underestimated (i.e., 43,800 on a global scale; 50.9% of correct answers). Substantial uncertainties were also reported on the current recommendations for mAb in clinical practice. For one, only 29.8% properly recognized pre-term infants as targeted individuals. Moreover, the commonly reported schedule for Palivizumab (i.e., 1 monthly dose during the RSV season; 38.6%), and their licensing only as a prophylactic option (43.7%) were extensively missed. Focusing on the role of natural immunity, the majority of participants (76.9%) properly addressed the potential preventive role of maternal antibodies, while only 53.2% of respondents acknowledged that RSV natural infections do not elicit a long-lasting immunity. 

### 3.3. Attitudes

**Figure 3:**
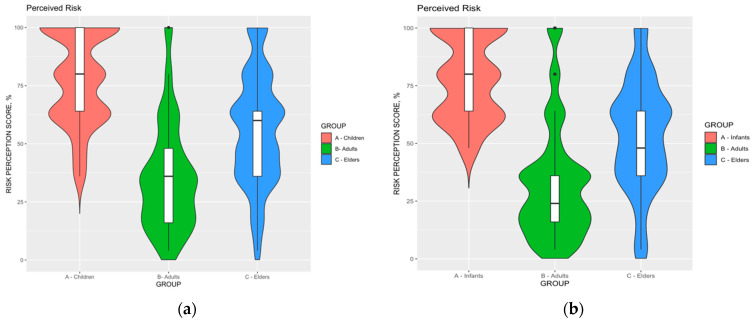
Violin plot with the comparisons of risk perception score (potential range: 0.0–100%) for Respiratory Syncytial Virus (RSV) infections in infants, adults, and elders. (**a**) All of the respondents’ (No. 389) RPS score for RSV infection in infants (78.3% ± 19.5, range 36.0% to 100%) were substantially higher than that for adults (35.5% ± 22.9, range 4.0% to 100; *p* < 0.001), and elders (56.1% ± 23.9, *p* < 0.001). (**b**) Only respondents having previously cared for RSV cases in previous 5 years (No. 163): RPS score for RSV infection in infants (78.3% ± 17.8, range 48.0% to 100%) was substantially higher than that for adults (32.7% ± 24.9, range 4.0% to 100; *p* < 0.001), and elders (53.6% ± 23.6, *p* < 0.001).

RSV was diffusely acknowledged as a somewhat common pathogen in infants (92.6%), with lower estimates for elders (54.8%), and adults (30.1%). A similar trend was associated with the perceived severity of RSV infections, as it was commonly acknowledged as high or very high in infants by the majority of respondents (84.9%), followed by elders (65.8%), being mostly overstated in adults (28.3%). As shown in Figure 3, corresponding estimates for RPS ranged from 78.3% ± 19.5 for infants (D’Agostino–Pearson *p* = 0.006), to 35.5% ± 22.9 in adults, while older age groups scored an intermediate estimate (56.1% ± 23.9; D’Agostino–Pearson *p* = 0.594). When a sub-analysis was performed by including only participants who had reported any previous interaction with RSV, the estimates for infants were highest (78.3% ± 17.8, range 48.0% to 100%), followed by those for elders (53.6% ± 23.6), and adults (32.7% ± 24.9, range 4.0% to 100%).

**Figure 4:**
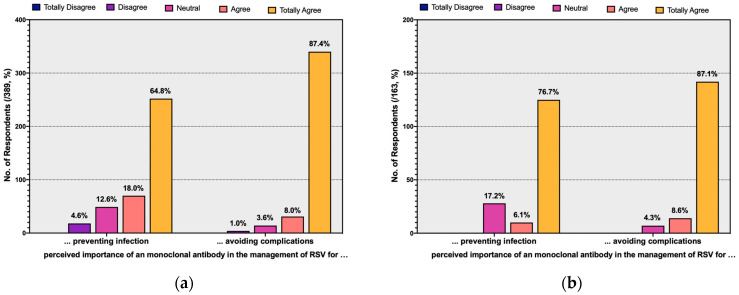
Ratings of the perceived importance of monoclonal antibodies in the management of respiratory tract infections by Respiratory Syncytial Virus (RSV) in terms of avoiding natural infections and its complications. Data are reported by including the sample as a whole (**a**), and only participants that had actually cared for RSV cases in the previous 5 years (**b**).

Interestingly, while the overwhelming majority of respondents (94.1%) exhibited some degree of acceptance of a tentative RSV vaccine when made commercially available, 74.8% of them were either favorable or highly favorable of the use of mAb. When participants were asked about the main features of a prophylactic therapy based on mAb, the majority of them either agreed or totally agreed on preventing complications (95.4%), followed by avoiding natural infection (82.8%). Similar proportions were also identified when a sub-analysis was performed on the respondents reporting any previous experience with RSV infections (95.7% and 82.8%, respectively) (Figure 4).

### 3.4. Previous Interactions with RSV

Among participants, 41.9% of them had previously managed at least one RSV case in the previous five years. Moreover, 34.4% did participate in the diagnosis, and 32.6% had required the eventual hospitalization of the patient because of LRTI. When focusing on the immunoprophylaxis with mAb, it had been previously recommended by 14.1% of participants.

### 3.5. Univariate Analysis

As shown in Figure 1, a greater estimate for GKS was identified among participants that had a previous professional experience with RSV cases (56.6% ± 15.5) than among those that had not (50.4% ± 12.6, *p* < 0.001). 

**Figure 5:**
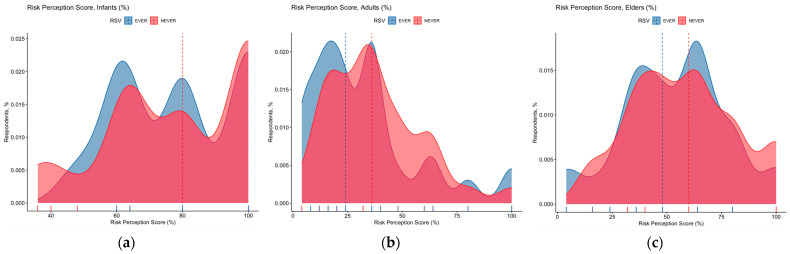
Density plot for Risk Perception Score (RPS) in infants (**a**), adults (**b**), and elderly (**c**) in 389 Italian Pediatricians participating into the survey. Cumulative scores were substantially skewed for infants (D’Agostino–Pearson’s normality test: *p* value 0.006) and adults (*p* < 0.001), but not for elderly (*p* = 0.594). When samples were broken down by having or not having previously cared for respiratory syncytial virus cases, no substantial differences in RPS were reported for infants (78.4% ± 17.8 vs. 78.3% ± 20.8; Mann–Whitney test *p* value 0.432), and elderly (53.6% ± 23.6 vs. 57.9% ± 23.9; *p* = 0.187). On the contrary, RPS for adults was substantially lower among participants having cared for RSV cases (32.7% ± 24.9) than among those having not (37.4% ± 21.3, *p* = 0.004).

Focusing on RPS estimates (Figure 5), RPS for infants was substantially greater than that reported for adults and elders, both in the sample as a whole and including only participants previously managing RSV cases (all comparisons, *p* < 0.001). On the contrary, RPS for adults was substantially lower among participants having cared for RSV cases (32.7% ± 24.9) than among those that had not (37.4% ± 21.3, *p* = 0.004), while no substantial differences were reported for infants (78.4% ± 17.8 vs. 78.3% ± 20.8; Mann–Whitney test *p* value 0.432), and elderly (53.6% ± 23.6 vs. 57.9% ± 23.9; *p* = 0.187).

GKS was only correlated with RPS for adults (rho = 0.155, *p* = 0.002). In turn, RPS for infants and elderly (rho = 0.174; *p* = 0.001), and adults and elderly (rho = 0.446, *p* < 0.001) were positively correlated (Table 2).

In univariate analysis for dichotomous variables (Table 3), having previously cared for RSV cases was negatively associated with a seniority ≥ 10 years (52.1% vs. 71.2%; *p* < 0.001), a higher RPS for adults (23.9% vs. 35.4%, *p* = 0.015), while it was positively associated with working in hospital settings (48.5% vs. 11.9%; *p* < 0.001). 

Table 4 shows the distribution of having or not having previously employed mAb for the prevention of RSV by demographic factors, risk perception status, knowledge status, and attitudes among participants who reportedly had any previous professional experience with RSV. Briefly, the previous use of mAb was less frequently reported among participants older than 40 years of age (30.4% vs. 47.7, *p* = 0.033), with greater seniority (42.9% vs. 47.0%, *p* = 0.086), of male gender (28.6% vs. 34.1%, *p* = 0.043), and from North-Eastern (23.2% vs. 40.2%) and Central Italy (21.4% vs. 34.6%). On the contrary, the use of mAb was more frequently reported among respondents from the Major Islands of Sicily and Sardinia (33.9% vs. 9.3%; *p* < 0.001), as well as among those working in hospital settings (64.3% vs. 30.2%), reporting higher GKS estimates (73.2% vs. 38.3%, *p* < 0.001), and scoring higher risk perception for infants (44.6% vs. 27.1%).

### 3.6. Regression Analysis

Regression analysis was therefore modelled as follows:

(a) Model 1 assessed the whole of the sample (i.e., 389 pediatricians) about the outcome variable of having had any previous experience in the managing of RSV cases, and assuming as explanatory variables: seniority ≥ 10 years, working in hospital settings; the region of residence; and reporting higher RPS for adults.
(b) Model 2 assessed all participants having reportedly managed any RSV case in the previous 5 years (i.e., 163 pediatricians). The analyses identified the previous delivery of mAb prophylactic therapy as the outcome variable, while the following explanatory variables were eventually included: belonging to an older age group (≥40 years); greater seniority (≥10 years); working in hospital settings; male gender; region of residence; higher GKS; and higher RPS for children.

The corresponding results are reported in Table 5. Briefly, having any previous experience with the managing of RSV cases was more frequently identified among professionals working in hospital settings (aOR 7.962; 95%CI 4.222 to 15.012). Moreover, when assuming the residence in North-Western Italy as a reference group, higher odds were scored for participants living in North-Eastern Italy (aOR 3.314, 95%CI 1.583 to 6.935), Central Italy (aOR 2.644; 95%CI 1.258 to 5.556), and Major Islands (aOR 14.373, 95%CI 4.861 to 42.498). 

Similarly, when the analyses were focused on participants having had any actual professional experience with RSV, having previously delivered mAb for RSV prophylaxis was positively associated with working in hospital settings (aOR 3.917, 95%CI 1.233 to 12.436), living in Major Islands (aOR 11.283, 95%CI 1.732 to 73.487), reporting higher GKS (aOR 33.933; 95%CI 7.746 to 148.457), and higher RPS on infants (aOR 7.295; 95%CI 1.977 to 26.924).

## 4. Discussion

In this cross-sectional study on a sample of Italian Pediatricians, the participants exhibited a largely favorable attitude towards the implementation of mAb as an instrument to prevent RSV complications. Interestingly, mAb were also extensively acknowledged as useful for avoiding natural infections, possibly overstating their actual efficacy [24,29,37,38,39,71]. However, despite this largely positive attitude, the sampled professionals reported a relatively infrequent use of mAb in their clinical practice: in fact, only 14.4% of the total sample, but also no more than 34.4% of the subgroup of participants with any previous expertise in the managing of RSV, had previously delivered mAb as a preventative item. In other words, even though the large majority of the participants exhibited a positive or even highly positive attitude towards mAb against RSV, only one third of the sampled pediatricians having reportedly managed any case of RSV had any expertise with these drugs. In our sample, both a higher familiarity on RSV and the previous use of mAb in the clinical practice for RSV prophylaxis were eventually associated with specific occupational features (i.e., working in the hospital settings), but also with other demographic characteristics such as the Italian region where the professional worked at the time of the survey. On the contrary, having any actual previous expertise in the use of mAb for RSV prophylaxis was associated with a better knowledge status and a higher risk perception for RSV infections in infants. 

Despite the increasing health burden associated with the post-lockdown resurgence of RSV [47,72,73,74], Public Health professionals are facing the long-lasting unavailability of reliable vaccines against this pathogen [75,76,77,78]. As promising as RSV vaccines actually are [79], by January 2023 no formulate has been made commercially available for childhood vaccination, and the two larger randomized controlled trials on maternal vaccinations still remain relatively far from their conclusion [80,81]. Although infant and maternal immunization programs could provide a potentially effective and long-lasting protection against RSV infections, mAb will therefore reasonably retain their clinical significance in the next years [5,52,75,76,77]. Collectively, such features could explain the favorable attitude towards an intervention that is perceived as both accessible and effective, as well as the substantial lack of familiarity with mAb in the clinical practice. Indeed, official recommendations for palivizumab are quite restrictive [10,16,27,29,30,31], prioritizing or even limiting the use of this drug on very selective groups of infants [34], and only after the conclusion of this survey’s extended half-life have mAb, such as nirsevimab, been available in the EU [35,36,38]. As nirsevimab’s easier and potentially more affordable handling could radically increase the share of targeted infants, it is quite reasonable that also the familiarity of clinical practitioners could increase in the next years [4,11,35,36,37,38,82].

When dealing with the effective predictors of the assessed outcomes, even though the Health Belief Model suggests that personal experiences and a rational understanding of a certain health topics (more precisely, an illness or a disease; in this case, RSV infections) and the beliefs in the effectiveness of a recommended health behavior or action will predict the likelihood of adopting that behavior (in this case, the delivery of mAb) [54,83,84,85,86,87,88], our results are only partially consistent, or even somewhat inconsistent with this underlying framework. 

On the one hand, the average estimates for GKS were far from optimal, with overall performances that were quite similar to our original study on general practitioners [51]. Even though individuals having previously cared for RSV scored better results than respondents that had not (i.e., 56.6% ± 15.5 vs. 50.4% ± 12.6, Mann–Whitney test *p* value < 0.001), a better knowledge status was not substantially associated with actual expertise of RSV cases. On the other hand, GKS and RPS were also not correlated, particularly when dealing with RPS on infants. In turn, a higher risk perception status on infant RSV infections was not substantially associated with reporting any personal experience in the management of RSV cases. Conversely, both knowledge status and risk perception on infants were characterized as strongly associated with having actually delivered a prophylactic therapy with mAb. Such seemingly inconsistent results hint towards a more complex interaction between knowledge status and actual practices that could be tentatively explained through the specific features of RSV infections in pediatric age. 

RSV is a very common pathogen, and a substantially number of infants develop an immune response within the second year or life [5,63,89]. In other words, while not interacting with any RSV infection during the ordinary pediatric practice is quite unlikely, being aware of the underlying diagnosis is a different story, particularly for primary care professionals [17,21,62]. As up to 97% of yearly incident cases of RSV infections are self-limited flu-like illnesses, the large majority of them are managed as outpatients until their recovery [90,91,92]. Therefore, even respondents with some previous experiences with RSV cases may underscore the actual significance of this infection [5,51], as well as its potential consequences in terms of long-lasting complications [53,57,93]. Conversely, as an appropriate microbiological diagnosis is limited to the severe cases of RSV-associated pneumonia or LRTI, that usually require more intensive care and even hospitalization [1,2,3,82], participants having any hospital background may perceive RSV infection as particularly common and severe. Similarly, it should be stressed that palivizumab (i.e., for nearly two decades the only commercially available mAb) is often delivered in hospital settings because of its direct and indirect costs, and the characteristics of the targeted infants [25,33,71]. As a consequence, not only higher familiarity with this class of drugs is somehow not unexpected among professionals having an occupational background in hospital settings, but they could even overstate the actual preventive significance of this class of drugs. In other words, it is both rational and reasonable that individuals having a greater familiarity with a pathogen that is often underrated in terms of potential severity and actual disease burden could eventually exhibit a better awareness of its actual features, as a well as a better attitude towards preventive options that are still uncommon in other settings [1,6,17,58,64,94,95,96,97,98]. In fact, not only participants that had cared for RSV cases were more frequently identified among professionals with a hospital background than among those that had not (aOR 7.962, 95%CI 4.222 to 15.012), but among this specific subgroup, not only hospital background (aOR 3.917; 95%CI 1.233 to 12.436) but also risk perception and knowledge status were characterized as effective predictors for the previous delivery of mAb (aOR 33.933; 95%CI 7.746 to 148.457, and aOR 7.295; 95%CI 1.977 to 26.924, respectively).

Even the somehow unexpected geographical trends could be explained through the heterogenous familiarity of the respondents with the pathogen and associated clinical features. The higher expertise with both RSV and mAb in participants from Major Islands of Sicily and Sardinia could be more easily understood by keeping in mind that Sicily (4.8 million inhabitants, 8.1% of Italian population) is characterized by a share of individuals aged 0–5 years that substantially exceeds national estimates (4.9% vs. 4.4%), with a higher share of households including two or more infants [99]. As RSV rather circulates among siblings than from parents and older adults to children, the actual circulation of the pathogen can be more easily understood from professionals working in these areas than in regions characterized by one-child households [5]. Moreover, a regional molecular surveillance system for RSV has also been implemented well before the inception of national programs [12,59,99,100,101]. Collectively, these factors may have diffusely increased the shared awareness on RSV infections, their complications, and the available preventative options. Therefore, our results not only provide some information on the KAP of medical professionals shortly before the official commercialization of new mAb, but stress the potential role of properly designed informative interventions for improving the understanding of RSV infections and the available preventive options, including the extended half-life mAb [52,102].

### Limits of This Study

Despite its potential significance, our study is affected by some substantial shortcomings. First and foremost, it shares all the conventional limits of Internet-based surveys, including the substantial “self-selection” of participants [65,103,104]. On the one hand, Internet-based studies usually oversample younger age groups, as characterized by subjects more familiar with the Internet and the social media that were employed for sharing the questionnaire [51,65,68,105]. Demographic features of the sample may in turn represent another critical issue, as we included a reduced share of respondents aged 50 year or older, and these figures are quite inconsistent with the rapidly aging Italian Medical workforce [106,107]. On the other hand, there is some evidence that web-based studies may similarly oversample individuals more interested and/or more familiar with the specifically assessed topic [54,103,108], a shortcoming that is otherwise shared with more conventional studies where the questionnaire is delivered by hand during a convenience event (e.g., meetings, professional courses, etc.) [54,108,109]. In order to cope with the potential self-selection of participants, our sampling strategy did prioritize a homogenous subgroup of medical professionals (in this case, pediatricians). However, we cannot rule out that some of the respondents did not fully adhere to our selection criteria. Sampling healthcare providers not managing infants and children would substantially compromise the actual representation of the sample, and the eventual reliability of our estimates. Even though the eventual sample size was consistent with our preventive estimates, the present study could only be cautiously generalizable, particularly in Italy, whose medical workforce is otherwise characterized by distinctive regional patterns, as stressed by our analyses [88].

Second, as for similarly designed KAP studies based on not-externally validated questionnaires, declarative bias, and particularly social desirability bias cannot be ruled out. Social desirability bias has been defined as the tendency of survey respondents to answer questions in a manner that will be viewed favorably by others [110]. As a topic, RSV has been associated with significant knowledge gaps not only in the general population [52], but also in caregivers [51,53,57,93], and the consequences of this shortcoming are potentially substantial, particularly among the sampled pediatricians having a limited experience in hospital settings, that may be unfamiliar with this pathogen [58,111,112]. As shown in Supplementary Table S3, even though knowledge status was seemingly similar between participants that did and did not have a hospital background, the latter had less frequently managed RSV cases (29.7% vs. 74.5%) and rarely recommended the delivery of mAb as a preventive option for RSV infections (7.1% vs. 34.0%). Not only knowledge status, but also the estimates on attitudes and beliefs of participants, may have similarly oversampled “common sense” of “more socially appropriate” answers on the actual attitudes of respondents [54,87,113,114]. In this regard, the RSV epidemic during winter season 2021–2022 has been characterized by an unprecedented media coverage. In order to ascertain the potential influence of media coverage on the shared beliefs and the eventual risk perception of respondents [4,51,99,115], a specific analysis was performed on the relative search volumes for RSV on Google Trends™. Google Trends^TM^ provides a normalized value ranging from 0 to 100, that is proportional to the ratio between the keyword-related queries and the total of web queries, representing a proxy of the general interest of the media on this topic [100,116,117]. As no significant correlation with RPS estimates and GKS was found (Table S4) [86], even though social desirability bias cannot be easily ruled out, media coverage had a reasonably limited impact on our estimates.

## 5. Conclusions

In conclusion, RSV was acknowledged as a common infection in all infants, and mAb were eventually characterized as an effective and reliable prophylactic option. Still, our study identified a substantial lack of personal expertise of the sampled professionals, not only on mAb, but also regarding RSV cases. Occupational background (i.e., working or having recently worked in hospital settings) possibly led the participants to a better understanding of RSV from a clinical and epidemiological point of view, as well as to a better familiarity with mAb-delivered preventive intervention. As a better knowledge status and a higher risk perception were in turn characterized as substantial effectors for delivering and having delivered mAb, a more specifically tailored formation of primary care providers could have the potential for increasing the acceptance and the actual use of these instruments by caregivers, ultimately improving their capability to cope with the needs of their patients. These interventions may be particularly useful as new and innovative mAb are progressively made available, enlarging the share of infants potentially benefiting from effective preventive interventions. 

## Figures and Tables

**Table 1:** Characteristics of the 389 Italian Pediatricians participating into the survey on knowledge, attitudes, and practices on respiratory syncytial virus.

Variable	No./389	Average ± SD
Gender		
Male	141, 36.2%	
Female	238, 61.2%	
Not stated	10, 2.6%	
Region		
North-Western Italy	69, 17.7%	
North-Eastern Italy	127, 32.6%	
Central Italy	122, 31.4%	
Southern Italy	35, 9.0%	
Major Islands	36, 9.3%	
Age (years)		40.1 ± 9.1
Seniority as PDL (years)		13.9 ± 9.0
Previously managed RSV cases	163, 41.9%	
Previously diagnosed RSV cases	134, 34.4%	
Previously required hospitalization for RSV	127, 32.6%	
Previously required mAb immunoprophylaxis for RSV	56, 14.4%	
Acknowledging RSV infection as frequent/very frequent in …		
… infants	360, 92.5%	
… adults	117, 30.1%	
… elderly	213, 54.8%	
Acknowledging RSV infection as severe/very severe in …		
… infants	330, 84.8%	
… adults	110, 28.3%	
… elderly	256, 65.8%	
General Knowledge Score (%)		54.0 ± 14.2
General Knowledge Score > median (52.0%)	179, 46.0%	
Risk Perception Score for infants		78.3 ± 19.5
Risk Perception Score for infants > median (80.0%)	142, 36.5%	
Risk Perception Score for adults		35.5 ± 22.9
Risk Perception Score for adults > median (36.0%)	119, 30.6%	
Risk Perception Score for elderly		56.1 ± 23.9
Risk Perception Score for elderly > median (60.0%)	187, 48.1%	
Favorable/Highly favorable to RSV vaccination when made available	366, 94.1%	
Attitude towards mAb (favorable/highly favorable)	291, 74.8%	
Acknowledging as significant/very significant aspects for mAb …		
… avoiding natural infection	322, 82.8%	
… avoiding complications (i.e., LRTI)	371, 95.4%	

**Table 2:** Correlation between General Knowledge Score (GKS), and the Risk Perception Scores (RPS) for Respiratory Syncytial Virus (RSV) infections in infants, adults, and elderly. Spearman’s correlation test (rho) with their respective *p* value.

Variable	GKS	RPS Infants	RPS Adults	RPS Elderly
GKS	-	0.021 (*p* = 0.676)	0.155 (*p* = 0.002)	−0.099 (*p* = 0.052)
RPS infants	0.021 (*p* = 0.676)	-	0.050 (*p* = 0.329)	0.174 (*p* = 0.001)
RPS adults	0.155 (*p* = 0.002)	0.050 (*p* = 0.329)	-	0.446 (*p* < 0.001)
RPS elderly	−0.099 (*p* = 0.052)	0.174 (*p* = 0.001)	0.446 (*p* < 0.001)	-

**Table 3:** Characteristics of the 389 Italian Pediatricians participating in the present survey on knowledge, attitudes, and practices on respiratory syncytial virus (RSV) by having or not having previously (i.e., in the previous 5 years) any cases of RSV in their practice.

Variable	Previously Managed RSV		*p* Value
	Ever (No./163, %)	Never (No./226, %)	
Age ≥ 40 years	68, 41.7%	102, 45.1%	0.503
Seniority ≥ 10 years	85, 52.1%	161, 71.2%	<0.001
Working in Hospital Settings	79, 48.5%	27, 11.9%	<0.001
Male Gender	64, 39.3%	77, 34.1%	0.293
Region of residence			<0.001
North-Western Italy	22, 13.5%	47, 20.8%	
North-Eastern Italy	56, 34.4%	71, 31.4%	
Central Italy	49, 30.1%	73, 32.3%	
Southern Italy	7, 4.3%	28, 12.4%	
Major Islands	29, 17.8%	7, 3.1%	
Higher Knowledge Status	81, 49.7%	129, 57.1%	0.149
Higher Risk Perception			
Infants	54, 33.1%	88, 38.9%	0.240
Adults	39, 23.9%	80, 35.4%	0.015
Elderly	78, 47.9%	109, 48.2%	0.941
Favorable attitude towards mAb	123, 75.5%	168, 74.3%	0.801

Interestingly, the status of having previously previous managed any RSV cases was more frequent among residents of Major Islands than having not (17.8% vs. 3.1%), and less frequent among those from North-Western Italy (13.5% vs. 20.0%; *p* < 0.001).

**Table 4:** Characteristics of the 163 Italian Pediatricians participating in the survey on knowledge, attitudes, and practices on respiratory syncytial (RSV) that in the 5 years before the present survey had managed any case of RSV by their referred use of monoclonal antibodies (mAb).

Variable	Previous Use of mAb		*p* Value
	Ever (No./56, %)	Never (No./107, %)	
Age ≥ 40 years	17, 30.4%	51, 47.7%	0.033
Seniority ≥ 10 years	24, 42.9%	61, 57.0%	0.086
Working in Hospital Settings	36, 64.3%	43, 40.2%	0.003
Male Gender	16, 28.6%	77, 34.1%	0.043
Region of residence			0.001
North-Western Italy	8, 14.3%	14, 13.1%	
North-Eastern Italy	13, 23.2%	43, 40.2%	
Central Italy	12, 21.4%	37, 34.6%	
Southern Italy	4, 7.1%	3, 2.8%	
Major Islands	19, 33.9%	10, 9.3%	
Higher Knowledge Status	41, 73.2%	41, 38.3%	<0.001
Higher Risk Perception			
Infants	25, 44.6%	29, 27.1%	0.024
Adults	13, 23.2%	26, 24.3%	0.877
Elderly	22, 39.3%	56, 52.3%	0.113
Attitude towards use of mAb	43, 76.8%	80, 74.8%	0.776

**Table 5:** Multivariable analysis of factors associated with (a) having previously (i.e., in the 5 years before the present survey) managed any respiratory syncytial virus (RSV) case (all respondents, No. 389; Model 1) and (b) having previously employed monoclonal antibodies targeting RSV as preventive therapy (only participants having previously managed a RSV case; No. 163). Adjusted Odds Ratios (adjOR) and their respective 95% confidence intervals were calculated through binary logistic regression analysis. In both models, all factors that in univariate analysis were associated with the outcome variables with *p* < 0.100 were included as explanatory variables.

Variable	Model 1		Model 2	
	aOR	95% CI	aOR	95% CI
Age ≥ 40 years	-	-	1.138	0.209; 6.200
Seniority ≥ 10 years	1.206	0.691; 2.103	0.412	0.066; 2.554
Working in Hospital Settings	7.962	4.222; 15.012	3.917	1.233; 12.436
Male Gender	-	-	0.168	0.054; 0.522
Region of residence				
North-Western Italy	1.000	REFERENCE	1.000	REFERENCE
North-Eastern Italy	3.314	1.583; 6.935	0.262	0.050; 1.375
Central Italy	2.644	1.258; 5.556	0.503	0.086; 2.941
Southern Italy	1.551	0.532; 4.526	1.099	0.111; 10.845
Major Islands	14.373	4.861; 42.498	11.283	1.732; 73.487
Higher Knowledge Status	-	-	33.933	7.756; 148.457
Higher Risk Perception				
Children	-	-	7.295	1.977; 26.924
Adults	0.632	0.364; 1.096	-	-

Notes: aOR = adjusted Odds Ratio (i.e., Odds Ratio calculated through binary logistic regression); 95%CI = 95% confidence interval.

## Data Availability

The data presented in this study are available on request from the corresponding author.

## Supplementary Materials

The following supporting information can be downloaded at: https://www.mdpi.com/article/10.3390/pediatric15010013/s1, Supplementary Material S1: Strobe statement Checklist; Supplementary Material S2: Author’s translation of the informed consent; Table S1: Authors’ translation of the items included in the questionnaire; Table S2. Knowledge test: summary of the answers on presented items proposed to the 389 Italian Pediatricians who participated into the present survey on Respiratory Syncytial Virus (RSV) (Cronbach’s alpha = 0.701). Table S3. Characteristics of the 389 Italian Pediatricians participating into the present survey on knowledge, attitudes and practices on respiratory syncytial virus (RSV) by working or not in hospital settings; Table S4. Correlation of relative search volumes (Google TrendsTM) on Respiratory Syncytial Virus (RSV) with General Knowledge Score (GKS) and Risk Perception Scores (RPS) (Spearman’s correlation coeffient).

## References

[1] Shi T., McAllister D.A., O’Brien K.L., Simoes E.A.F., Madhi S.A., Gessner B.D., Polack F.P., Balsells E., Acacio S., Aguayo C. (2017). Global, Regional, and National Disease Burden Estimates of Acute Lower Respiratory Infections Due to Respiratory Syncytial Virus in Young Children in 2015: A Systematic Review and Modelling Study. Lancet.

[2] Shi T., Denouel A., Tietjen A.K., Campbell I., Moran E., Li X., Campbell H., Demont C., Nyawanda B.O., Chu H.Y. (2021). Global Disease Burden Estimates of Respiratory Syncytial Virus-Associated Acute Respiratory Infection in Older Adults in 2015: A Systematic Review and Meta-Analysis. J. Infect. Dis..

[3] Li Y., Wang X., Blau D.M., Caballero M.T., Feikin D.R., Gill C.J., Madhi S.A., Omer S.B., Simões E.A.F., Campbell H. (2022). Global, Regional, and National Disease Burden Estimates of Acute Lower Respiratory Infections Due to Respiratory Syncytial Virus in Children Younger than 5 Years in 2019: A Systematic Analysis. Lancet.

[4] Esposito S., Abu Raya B., Baraldi E., Flanagan K., Martinon Torres F., Tsolia M., Zielen S. (2022). RSV Prevention in All Infants: Which Is the Most Preferable Strategy?. Front. Immunol..

[5] Baraldi E., Checcucci Lisi G., Costantino C., Heinrichs J.H., Manzoni P., Riccò M., Roberts M., Vassilouthis N. (2022). RSV Disease in Infants and Young Children: Can We See a Brighter Future?. Hum. Vaccines Immunother..

[6] Nair H., Nokes D.J., Gessner B.D., Dherani M., Madhi S.A., Singleton R.J., O’Brien K.L., Roca A., Wright P.F., Bruce N. (2010). Global Burden of Acute Lower Respiratory Infections Due to Respiratory Syncytial Virus in Young Children: A Systematic Review and Meta-Analysis. Lancet.

[7] Leader S., Kohlhase K. (2002). Respiratory syncytial virus-coded pediatric hospitalizations, 1997 to 1999. Pediatr. Infect. Dis. J..

[8] Na’Amnih W., Kassem E., Tannous S., Kagan V., Jbali A., Hanukayev E., Freimann S., Obolski U., Muhsen K. (2022). Incidence and risk factors of hospitalisations for respiratory syncytial virus among children aged less than 2 years. Epidemiology Infect..

[9] Jans J., Wicht O., Widjaja I., Ahout I.M.L., de Groot R., Guichelaar T., Luytjes W., de Jonge M.I., de Haan C.A.M., Ferwerda G. (2017). Characteristics of RSV-Specific Maternal Antibodies in Plasma of Hospitalized, Acute RSV Patients under Three Months of Age. PLoS ONE.

[10] Chida-Nagai A., Sato H., Sato I., Shiraishi M., Sasaki D., Izumi G., Yamazawa H., Cho K., Manabe A., Takeda A. (2021). Risk factors for hospitalisation due to respiratory syncytial virus infection in children receiving prophylactic palivizumab. Eur. J. Pediatr..

[11] Azzari C., Baraldi E., Bonanni P., Bozzola E., Coscia A., Lanari M., Manzoni P., Mazzone T., Sandri F., Lisi G.C. (2021). Epidemiology and prevention of respiratory syncytial virus infections in children in Italy. Ital. J. Pediatr..

[12] Pellegrinelli L., Galli C., Bubba L., Cereda D., Anselmi G., Binda S., Gramegna M., Pariani E. (2020). Respiratory syncytial virus in influenza-like illness cases: Epidemiology and molecular analyses of four consecutive winter seasons (2014-2015/2017-2018) in Lombardy (Northern Italy). J. Med. Virol..

[13] Openshaw P.J., Chiu C., Culley F.J., Johansson C. (2017). Protective and Harmful Immunity to RSV Infection. Annu. Rev. Immunol..

[14] Andeweg S.P., Schepp R.M., van de Kassteele J., Mollema L., Berbers G.A.M., van Boven M. (2021). Population-based serology reveals risk factors for RSV infection in children younger than 5 years. Sci. Rep..

[15] Mazur N.I., Martinón-Torres F., Baraldi E., Fauroux B., Greenough A., Heikkinen T., Manzoni P., Mejias A., Nair H., Papadopoulos N.G. (2015). Lower respiratory tract infection caused by respiratory syncytial virus: Current management and new therapeutics. Lancet Respir. Med..

[16] Griffiths C., Drews S.J., Marchant D.J. (2017). Respiratory Syncytial Virus: Infection, Detection, and New Options for Prevention and Treatment. Clin. Microbiol. Rev..

[17] Falsey A.R., Hennessey P.A., Formica M.A., Cox C., Walsh E.E. (2005). Respiratory Syncytial Virus Infection in Elderly and High-Risk Adults. N. Engl. J. Med..

[18] Giersing B.K., Karron R.A., Vekemans J., Kaslow D.C., Moorthy V.S. (2019). Meeting report: WHO consultation on Respiratory Syncytial Virus (RSV) vaccine development, Geneva, 25–26 April 2016. Vaccine.

[19] Mosalli R., Alqarni S.A., Khayyat W.W., Alsaidi S.T., Almatrafi A.S., Bawakid A.S., Paes B. (2021). Respiratory syncytial virus nosocomial outbreak in neonatal intensive care: A review of the incidence, management, and outcomes. Am. J. Infect. Control..

[20] Ruckwardt T.J., Morabito K.M., Graham B.S. (2019). Immunological Lessons from Respiratory Syncytial Virus Vaccine Development. Immunity.

[21] Debes S., Haug J.B., de Blasio B.F., Jonassen C.M., Dudman S.G. (2021). Etiology of viral respiratory tract infections in hospitalized adults, and evidence of the high frequency of prehospitalization antibiotic treatment in Norway. Health Sci. Rep..

[22] Obolski U., Kassem E., Na’Amnih W., Tannous S., Kagan V., Muhsen K. (2021). Unnecessary antibiotic treatment of children hospitalised with respiratory syncytial virus (RSV) bronchiolitis: Risk factors and prescription patterns. J. Glob. Antimicrob. Resist..

[23] Andabaka T., Nickerson J.W., Rojas-Reyes M.X., Rueda J.D., Vrca V.B., Barsic B. (2013). Monoclonal antibody for reducing the risk of respiratory syncytial virus infection in children. Cochrane Database Syst. Rev..

[24] Frogel M.P., Stewart D.L., Hoopes M., Fernandes A.W., Mahadevia P.J. (2010). A Systematic Review of Compliance with Palivizumab Administration for RSV Immunoprophylaxis. J. Manag. Care Pharm..

[25] Olchanski N., Hansen R.N., Pope E., D’Cruz B., Fergie J., Goldstein M., Krilov L.R., McLaurin K.K., Nabrit-Stephens B., Oster G. (2018). Palivizumab Prophylaxis for Respiratory Syncytial Virus: Examining the Evidence Around Value. Open Forum Infect. Dis..

[26] Mac S., Sumner A., Duchesne-Belanger S., Stirling R., Tunis M., Sander B. (2019). Cost-effectiveness of Palivizumab for Respiratory Syncytial Virus: A Systematic Review. Pediatrics.

[27] Viguria N., Navascués A., Juanbeltz R., Echeverría A., Ezpeleta C., Castilla J. (2021). Effectiveness of palivizumab in preventing respiratory syncytial virus infection in high-risk children. Hum. Vaccines Immunother..

[28] Luna M.S., Manzoni P., Paes B., Baraldi E., Cossey V., Kugelman A., Chawla R., Dotta A., Fernández R.R., Resch B. (2018). Expert consensus on palivizumab use for respiratory syncytial virus in developed countries. Paediatr. Respir. Rev..

[29] Mitchell I., Li A., Bjornson C.L., Lanctot K.L., Paes B.A., the CARESS investigators (2021). Respiratory Syncytial Virus Immunoprophylaxis with Palivizumab: 12-Year Observational Study of Usage and Outcomes in Canada. Am. J. Perinatol..

[30] Zylbersztejn A., Almossawi O., Gudka N., Tompsett D., De Stavola B., Standing J.F., Smyth R., Hardelid P. (2021). Access to palivizumab among children at high risk of respiratory syncytial virus complications in English hospitals. Br. J. Clin. Pharmacol..

[31] Batista J.D.L., Ferreira M.A.P., Xavier C.D.S., de Souza I.T.A., Cruz L.N., Polanczyk C.A. (2021). A post-incorporation study on the use of palivizumab in the Brazilian public health system. Rev. Do Inst. De Med. Trop. De São Paulo.

[32] Meissner H.C., Long S.S. (2003). Committee on Infectious Diseases and Committee on Fetus and Newborn Revised Indications for the Use of Palivizumab and Respiratory Syncytial Virus Immune Globulin Intravenous for the Prevention of Respiratory Syncytial Virus Infections. Pediatrics.

[33] Cutrera R., Wolfler A., Picone S., Rossi G.A., Gualberti G., Merolla R., Del Vecchio A., Villani A., Midulla F., Dotta A. (2019). Impact of the 2014 American Academy of Pediatrics recommendation and of the resulting limited financial coverage by the Italian Medicines Agency for palivizumab prophylaxis on the RSV-associated hospitalizations in preterm infants during the 2016–2017 epidemic season: A systematic review of seven Italian reports. Ital. J. Pediatr..

[34] Brady M.T., Byington C.L., Dele Davies H., Edwards K.M., Jackson M.A., Maldonado Y.A., Murray D.L., Orenstein W.A., American Academy of Pediatrics Committee on Infectious Diseases, American Academy of Pediatrics Bronchiolitis Guidelines Committee (2014). Updated Guidance for Palivizumab Prophylaxis Among Infants and Young Children at Increased Risk of Hospitalization for Respiratory Syncytial Virus Infection. Pediatrics.

[35] Hammitt L.L., Dagan R., Yuan Y., Cots M.B., Bosheva M., Madhi S.A., Muller W.J., Zar H.J., Brooks D., Grenham A. (2022). Nirsevimab for Prevention of RSV in Healthy Late-Preterm and Term Infants. New Engl. J. Med..

[36] Griffin M.P., Yuan Y., Takas T., Domachowske J.B., Madhi S.A., Manzoni P., Simões E.A.F., Esser M.T., Khan A.A., Dubovsky F. (2020). Single-Dose Nirsevimab for Prevention of RSV in Preterm Infants. N. Engl. J. Med..

[37] Voirin N., Virlogeux V., Demont C., Kieffer A. (2021). Potential Impact of Nirsevimab on RSV Transmission and Medically Attended Lower Respiratory Tract Illness Caused by RSV: A Disease Transmission Model. Infect. Dis. Ther..

[38] Domachowske J., Madhi S.A., Simões E.A., Atanasova V., Cabañas F., Furuno K., Garcia-Garcia M.L., Grantina I., Nguyen K.A., Brooks D. (2022). Safety of Nirsevimab for RSV in Infants with Heart or Lung Disease or Prematurity. New Engl. J. Med..

[39] Bergeron H.C., Tripp R.A. (2021). Breakthrough therapy designation of nirsevimab for the prevention of lower respiratory tract illness caused by respiratory syncytial virus infections (RSV). Expert Opin. Investig. Drugs.

[40] Domachowske J.B., Anderson E.J., Goldstein M. (2021). The Future of Respiratory Syncytial Virus Disease Prevention and Treatment. Infect. Dis. Ther..

[41] Calderaro A., De Conto F., Buttrini M., Piccolo G., Montecchini S., Maccari C., Martinelli M., Di Maio A., Ferraglia F., Pinardi F. (2020). Human respiratory viruses, including SARS-CoV-2, circulating in the winter season 2019–2020 in Parma, Northern Italy. Int. J. Infect. Dis..

[42] Sherman A.C., Babiker A., Sieben A.J., Pyden A., Steinberg J., Kraft C.S., Koelle K., Kanjilal S. (2020). The Effect of Severe Acute Respiratory Syndrome Coronavirus 2 (SARS-CoV-2) Mitigation Strategies on Seasonal Respiratory Viruses: A Tale of 2 Large Metropolitan Centers in the United States. Clin. Infect. Dis..

[43] Kuitunen I.M., Artama M.M., Mäkelä L., Backman K.M., Heiskanen-Kosma T.M., Renko M.M. (2020). Effect of Social Distancing Due to the COVID-19 Pandemic on the Incidence of Viral Respiratory Tract Infections in Children in Finland During Early 2020. Pediatr. Infect. Dis. J..

[44] Van Brusselen D., de Troeyer K., ter Haar E., van der Auwera A., Poschet K., van Nuijs S., Bael A., Stobbelaar K., Verhulst S., van Herendael B. (2021). Bronchiolitis in COVID-19 Times: A Nearly Absent Disease?. Eur. J. Pediatr..

[45] Britton P.N., Hu N., Saravanos G., Shrapnel J., Davis J., Snelling T., Dalby-Payne J., Kesson A.M., Wood N., Macartney K. (2020). COVID-19 public health measures and respiratory syncytial virus. Lancet Child Adolesc. Health.

[46] Ninot G., Ninot G. (2021). Defining Non-Pharmacological Interventions. Non-Pharmacological Interventions an Essential Answer to Current Demographic, Health, and Environmental Transitions.

[47] Hatter L., Eathorne A., Hills T., Bruce P., Beasley R. (2021). Respiratory syncytial virus: Paying the immunity debt with interest. Lancet Child Adolesc. Health.

[48] Foley D.A., Phuong L.K., Peplinski J., Lim S.M., Lee W.H., Farhat A., Minney-Smith C.A., Martin A.C., Mace A.O., Sikazwe C.T. (2021). Examining the interseasonal resurgence of respiratory syncytial virus in Western Australia. Arch. Dis. Child..

[49] Foley D.A., Yeoh D.K., Minney-Smith C.A., Martin A.C., Mace A.O., Sikazwe C.T., Le H., Levy A., Moore H.C., Blyth C.C. (2021). The Interseasonal Resurgence of Respiratory Syncytial Virus in Australian Children Following the Reduction of Coronavirus Disease 2019—Related Public Health Measures. Clin. Infect. Dis..

[50] Lumley S.F., Richens N., Lees E., Cregan J., Kalimeris E., Oakley S., Morgan M., Segal S., Dawson M., Walker A.S. (2021). Changes in pediatric respiratory infections at a UK teaching hospital 2016–2021; impact of the SARS-CoV-2 pandemic. J. Infect..

[51] Riccò M., Ferraro P., Peruzzi S., Zaniboni A., Ranzieri S. (2022). Respiratory Syncytial Virus: Knowledge, Attitudes and Beliefs of General Practitioners from North-Eastern Italy (2021). Pediatr. Rep..

[52] Giles M.L., Buttery J., Davey M.-A., Wallace E. (2019). Pregnant women’s knowledge and attitude to maternal vaccination including group B streptococcus and respiratory syncytial virus vaccines. Vaccine.

[53] Wilcox C.R., Calvert A., Metz J., Kilich E., MacLeod R., Beadon K., Heath P.T., Khalil A., Finn A., Snape M.D. (2019). Attitudes of Pregnant Women and Healthcare Professionals Toward Clinical Trials and Routine Implementation of Antenatal Vaccination Against Respiratory Syncytial Virus: A Multicenter Questionnaire Study. Pediatr. Infect. Dis. J..

[54] Betsch C., Wicker S. (2014). Personal attitudes and misconceptions, not official recommendations guide occupational physicians’ vaccination decisions. Vaccine.

[55] Von Elm E., Altman D.G., Egger M., Pocock S.J., Gøtsche P.C., Vandenbroucke J.P., STROBE Initiative (2007). Strengthening the Reporting of Observational Studies in Epidemiology (StroBE) Statement: Guidelines for Reporting Observational Studies. BMJ.

[56] Cecchini E., Schino S., Gambadoro N., Ricciardi L., Trio O., Biondi-Zoccai G., Sangiorgi G. (2022). Facing the pandemic with a smile: The case of Memedical and its impact on cardiovascular professionals. Minerva Cardioangiol..

[57] Hurley L.P., Allison M.A., Kim L., O’Leary S.T., Crane L.A., Brtnikova M., Beaty B.L., Allen K.E., Poser S., Lindley M.C. (2018). Primary care physicians’ perspectives on respiratory syncytial virus (RSV) disease in adults and a potential RSV vaccine for adults. Vaccine.

[58] Reeves R., Hardelid P., Panagiotopoulos N., Minaji M., Warburton F., Pebody R. (2019). Burden of hospital admissions caused by respiratory syncytial virus (RSV) in infants in England: A data linkage modelling study. J. Infect..

[59] Barbati F., Moriondo M., Pisano L., Calistri E., Lodi L., Ricci S., Giovannini M., Canessa C., Indolfi G., Azzari C. (2020). Epidemiology of Respiratory Syncytial Virus-Related Hospitalization Over a 5-Year Period in Italy: Evaluation of Seasonality and Age Distribution Before Vaccine Introduction. Vaccines.

[60] Rainisch G., Adhikari B., Meltzer M.I., Langley G. (2019). Estimating the impact of multiple immunization products on medically-attended respiratory syncytial virus (RSV) infections in infants. Vaccine.

[61] Palmer L., Hall C.B., Katkin J.P., Shi N., Masaquel A.S., McLaurin K.K., Mahadevia P.J. (2010). Healthcare costs within a year of respiratory syncytial virus among medicaid infants. Pediatr. Pulmonol..

[62] Shi T., Arnott A., Semogas I., Falsey A.R., Openshaw P., Wedzicha J.A., Campbell H., Nair H., Zhang S., Li Y. (2019). The Etiological Role of Common Respiratory Viruses in Acute Respiratory Infections in Older Adults: A Systematic Review and Meta-analysis. J. Infect. Dis..

[63] Staadegaard L., Caini S., Wangchuk S., Thapa B., de Almeida W.A.F., de Carvalho F.C., Njouom R., A Fasce R., Bustos P., Kyncl J. (2021). The Global Epidemiology of RSV in Community and Hospitalized Care: Findings From 15 Countries. Open Forum Infect. Dis..

[64] Ali A., Lopardo G., Scarpellini B., Stein R.T., Ribeiro D. (2019). Systematic review on respiratory syncytial virus epidemiology in adults and the elderly in Latin America. Int. J. Infect. Dis..

[65] Riccò M., Gualerzi G., Ranzieri S., Ferraro P., Bragazzi N. (2020). Knowledge, Attitudes, Practices (KAP) of Italian Occupational Physicians towards Tick Borne Encephalitis. Trop. Med. Infect. Dis..

[66] Riccò M., Razio B., Panato C., Poletti L., Signorelli C. (2017). Knowledge, Attitudes and Practices of Agricultural Workers towards Tetanus Vaccine: A Field Report. Ann Ig.

[67] Riccò M., Ferraro P., Peruzzi S., Balzarini F., Ranzieri S. (2021). Mandate or Not Mandate: Knowledge, Attitudes, and Practices of Italian Occupational Physicians towards SARS-CoV-2 Immunization at the Beginning of Vaccination Campaign. Vaccines.

[68] Riccò M., Ferraro P., Camisa V., Satta E., Zaniboni A., Ranzieri S., Baldassarre A., Zaffina S., Marchesi F. (2022). When a Neglected Tropical Disease Goes Global: Knowledge, Attitudes and Practices of Italian Physicians towards Monkeypox, Preliminary Results. Trop. Med. Infect. Dis..

[69] Yates F.J., Stone E.R., Yates F.J. (1992). The Risk Construct. Risk-Taking Behaviour.

[70] R Core Team (2013). R: A Language and Environment for Statistical Computing.

[71] Weiner J.H. (2009). Respiratory Syncytial Virus Infection and Palivizumab: Are Families Receiving Accurate Information?. Am. J. Perinatol..

[72] Rybak A., Levy C., Jung C., Béchet S., Batard C., Hassid F., Zouari M., Cahn-Sellem F., Bangert M., Cohen R. (2021). Delayed Bronchiolitis Epidemic in French Primary Care Setting Driven by Respiratory Syncytial Virus: Preliminary Data from the Oursyn Study, March 2021. Pediatr. Infect. Dis. J..

[73] Bozzola E., Ciarlitto C., Guolo S., Brusco C., Cerone G., Antilici L., Schettini L., Piscitelli A.L., Vittucci A.C., Cutrera R. (2021). Respiratory Syncytial Virus Bronchiolitis in Infancy: The Acute Hospitalization Cost. Front. Pediatr..

[74] Di Mattia G., Nenna R., Mancino E., Rizzo V., Pierangeli A., Villani A., Midulla F. (2021). During the COVID-19 pandemic where has respiratory syncytial virus gone?. Pediatr. Pulmonol..

[75] Biagi C., Dondi A., Scarpini S., Rocca A., Vandini S., Poletti G., Lanari M. (2020). Current State and Challenges in Developing Respiratory Syncytial Virus Vaccines. Vaccines.

[76] Gunatilaka A., Giles M.L. (2021). Maternal RSV Vaccine Development. Where to from Here?. Hum. Vaccines Immunother..

[77] Graham B.S. (2017). Vaccine development for respiratory syncytial virus. Curr. Opin. Virol..

[78] Mejias A., Rodríguez-Fernández R., Oliva S., Peeples M.E., Ramilo O. (2020). The journey to a respiratory syncytial virus vaccine. Ann. Allergy Asthma Immunol..

[79] Ginsburg A.S., Srikantiah P. (2021). Respiratory syncytial virus: Promising progress against a leading cause of pneumonia. Lancet Glob. Health.

[80] ClinicalTrials.gov (2022). A Trial to Evaluate the Efficacy and Safety of RSVpreF in Infants Born to Women Vaccinated During Pregnancy. https://clinicaltrials.gov/ct2/show/NCT04424316.

[81] ClinicalTrials.gov (2022). A phase III Double-Blind Study to Assess Safety and Efficacy of an RSV Maternal Unadjuvanted Vaccine, in Pregnant Women and Infants Born to Vaccinated Mothers (GRACE). https://clinicaltrials.gov/ct2/show/results/NCT04605159.

[82] Baraldi E., Lanari M., Manzoni P., Rossi G.A., Vandini S., Rimini A., Romagnoli C., Colonna P., Biondi A., Biban P. (2014). Inter-society consensus document on treatment and prevention of bronchiolitis in newborns and infants. Ital. J. Pediatr..

[83] Janz N.K., Becker M.H. (1984). The Health Belief Model: A Decade Later. Health Educ. Q..

[84] Rosenstock I.M. (1974). Historical Origins of the Health Belief Model. Health Educ. Monogr..

[85] Carpenter C.J. (2010). A Meta-Analysis of the Effectiveness of Health Belief Model Variables in Predicting Behavior. Health Commun..

[86] Mo P.K.H., Lau J.T.F. (2015). Influenza vaccination uptake and associated factors among elderly population in Hong Kong: The application of the Health Belief Model. Health Educ. Res..

[87] Riccò M., Cattani S., Casagranda F., Gualerzi G., Signorelli C. (2017). Knowledge, attitudes, beliefs and practices of occupational physicians towards vaccinations of health care workers: A cross sectional pilot study in North-Eastern Italy. Int. J. Occup. Med. Environ. Health.

[88] Riccò M., Peruzzi S. (2022). Tetanus Vaccination Status and Vaccine Hesitancy in Amateur Basketball Players (Italy, 2020). Vaccines.

[89] Begley K.M., Leis A.M., Petrie J.G., Johnson E., McSpadden E., Lamerato L.E., Wei M., Martin E.T. (2022). Epidemiology of RSV-A and RSV-B in Adults and Children with Medically-Attended Acute Respiratory Illness over Three Seasons. medRxiv.

[90] Lively J.Y., Curns A.T., Weinberg G.A., Edwards K.M., Staat M.A., Prill M.M., Gerber S.I., Langley G.E. (2019). Respiratory syncytial virus–associated outpatient visits among children younger than 24 months. J. Pediatr. Infect. Dis. Soc..

[91] Arriola C.S., Kim L., Langley G., Anderson E.J., Openo K., Martin A.M., Lynfield R., Bye E., Como-Sabetti K., Reingold A. (2019). Estimated Burden of Community-Onset Respiratory Syncytial Virus–Associated Hospitalizations Among Children Aged <2 Years in the United States, 2014–2015. J. Pediatr. Infect. Dis. Soc..

[92] Byington C.L., Wilkes J., Korgenski K., Sheng X. (2015). Respiratory Syncytial Virus–Associated Mortality in Hospitalized Infants and Young Children. Pediatrics.

[93] Allen K.E., Beekmann S.E., Polgreen P., Poser S., Pierre J.S., Santibañez S., Gerber S.I., Kim L. (2018). Survey of diagnostic testing for respiratory syncytial virus (RSV) in adults: Infectious disease physician practices and implications for burden estimates. Diagn. Microbiol. Infect. Dis..

[94] Hall C.B., Weinberg G.A., Iwane M.K., Blumkin A.K., Edwards K.M., Staat M.A., Auinger P., Griffin M.R., Poehling K.A., Erdman D. (2009). The Burden of Respiratory Syncytial Virus Infection in Young Children. N. Engl. J. Med..

[95] Demont C., Petrica N., Bardoulat I., Duret S., Watier L., Chosidow A., Lorrot M., Kieffer A., Lemaitre M. (2021). Economic and disease burden of RSV-associated hospitalizations in young children in France, from 2010 through 2018. BMC Infect. Dis..

[96] Auvinen R., Syrjänen R., Ollgren J., Nohynek H., Skogberg K. (2021). Clinical characteristics and population-based attack rates of respiratory syncytial virus versus influenza hospitalizations among adults—An observational study. Influ. Other Respir. Viruses.

[97] Yung C.-F., Lee K.-S., Thein T.-L., Tan L.-K., Gan V., Wong J., Lye D., Ng L.-C., Leo Y.-S. (2015). Dengue Serotype-Specific Differences in Clinical Manifestation, Laboratory Parameters and Risk of Severe Disease in Adults, Singapore. Am. J. Trop. Med. Hyg..

[98] Hall C.B., Long C.E., Schnabel K.C. (2001). Respiratory Syncytial Virus Infections in Previously Healthy Working Adults. Clin. Infect. Dis..

[99] Tramuto F., Maida C.M., Di Naro D., Randazzo G., Vitale F., Restivo V., Costantino C., Amodio E., Casuccio A., Graziano G. (2021). Respiratory Syncytial Virus: New Challenges for Molecular Epidemiology Surveillance and Vaccination Strategy in Patients with ILI/SARI. Vaccines.

[100] Riccò M., Baldassarre A., Provenzano S., Corrado S., Cerviere M.P., Parisi S., Marchesi F., Bottazzoli M. (2022). Infodemiology of RSV in Italy (2017–2022): An Alternative Option for the Surveillance of Incident Cases in Pediatric Age?. Children.

[101] Pariani E., Amendola A., Piatti A., Anselmi G., Ranghiero A., Bubba L., Rosa A.M., Pellegrinelli L., Binda S., Coppola L. (2015). Ten Years (2004–2014) of Influenza Surveillance in Northern Italy. Hum. Vaccines Immunother..

[102] Mortensen G.L., Harrod-Lui K. (2022). Parental knowledge about respiratory syncytial virus (RSV) and attitudes to infant immunization with monoclonal antibodies. Expert Rev. Vaccines.

[103] Heiervang E., Goodman R. (2009). Advantages and limitations of web-based surveys: Evidence from a child mental health survey. Soc. Psychiatry Psychiatr. Epidemiol..

[104] Huang Y., Xu S., Wang L., Zhao Y., Liu H., Yao D., Xu Y., Lv Q., Hao G., Xu Y. (2017). Knowledge, Attitudes, and Practices Regarding Zika: Paper- and Internet-Based Survey in Zhejiang, China. JMIR Public Health Surveill..

[105] Riccò M., Ferraro P., Camisa V., Di Palma P., Minutolo G., Ranzieri S., Zaffina S., Baldassarre A., Restivo V. (2022). Managing of Migraine in the Workplaces: Knowledge, Attitudes and Practices of Italian Occupational Physicians. Medicina.

[106] Riccò M., Vezzosi L., Balzarini F. (2020). Challenges faced by the Italian medical workforce. Lancet.

[107] Vicarelli G., Pavolini E. (2015). Health workforce governance in Italy. Health Policy.

[108] Riccò M., Vezzosi L., Gualerzi G., Balzarini F., A Capozzi V., Volpi L. (2019). Knowledge, attitudes, beliefs and practices of obstetrics-gynecologists on seasonal influenza and pertussis immunizations in pregnant women: Preliminary results from North-Western Italy. Minerva Obstet. Gynecol..

[109] Zingg A., Siegrist M. (2012). Measuring people’s knowledge about vaccination: Developing a one-dimensional scale. Vaccine.

[110] Krumpal I. (2011). Determinants of social desirability bias in sensitive surveys: A literature review. Qual. Quant..

[111] Sharp A., Minaji M., Panagiotopoulos N., Reeves R., Charlett A., Pebody R. (2021). Estimating the burden of adult hospital admissions due to RSV and other respiratory pathogens in England. Influ. Other Respir. Viruses.

[112] Cromer D., van Hoek A.J., Newall A., Pollard A.J., Jit M. (2017). Burden of paediatric respiratory syncytial virus disease and potential effect of different immunisation strategies: A modelling and cost-effectiveness analysis for England. Lancet Public Health.

[113] Riccò M., Cattani S., Casagranda F., Gualerzi G., Signorelli C. (2017). Knowledge, attitudes, beliefs and practices of Occupational Physicians towards seasonal influenza vaccination: A cross-sectional study from North-Eastern Italy. J. Prev. Med. Hyg..

[114] Riccò M., Vezzosi L., Gualerzi G., Bragazzi N.L., Balzarini F. (2020). Pertussis immunization in healthcare workers working in pediatric settings: Knowledge, Attitudes and Practices (KAP) of Occupational Physicians. Preliminary results from a web-based survey (2017). J. Prev. Med. Hyg..

[115] Bozzola E. (2021). Respiratory Syncytial Virus Resurgence in Italy: The Need to Protect All Neonates and Young Infants. Int. J. Environ. Res. Public Health.

[116] Rovetta A. (2021). Reliability of Google Trends: Analysis of the Limits and Potential of Web Infoveillance During COVID-19 Pandemic and for Future Research. Front. Res. Metrics Anal..

[117] Riccò M., Zaniboni A., Satta E., Ranzieri S., Cerviere M.P., Marchesi F., Peruzzi S. (2022). West Nile Virus Infection: A Cross-Sectional Study on Italian Medical Professionals during Summer Season 2022. Trop. Med. Infect. Dis..

