# Supplementary material for: Respiratory Syncytial Virus Prevention through Monoclonal Antibodies: A Cross-Sectional Study on Knowledge, Attitudes, and Practices of Italian Pediatricians
Source: Pediatr Rep. 2023 Feb 20;15(1):154–74. doi: 10.3390/pediatric15010013 (PMC9944855; doi:10.3390/pediatric15010013)
Supplement: Supplementary file 1 [file pediatrrep-15-00013-s001.zip › pediatrrep-1961742-Supplementary Tables S1-S4.pdf]

Supplementary Tables

Table S1. Authors' translation of the items included in the questionnaire.

**Section 1. Your personal experience with RSV infections: during your clinical practice …**	
Have you previously managed any RSV case?	[YES] [NO][NO ANSWER]
Have you previously diagnosed any RSV case?	[YES] [NO][NO ANSWER]
Have previously required any hospitalization for RSV?	[YES] [NO][NO ANSWER]
Have you previously required mAb immunoprophylaxis for RSV?	[YES] [NO][NO ANSWER]
**Section 2. At your knowledge (please mark the correct answer)**	
1. Nearly all RSV infections occur in infants and children	[TRUE] [FALSE] [DON'T KNOW]
2. In most cases, infants acquire RSV infections from their parents	[TRUE] [FALSE] [DON'T KNOW]
3. In most cases, RSV evolves in an uncomplicated influenza-like illness	[TRUE] [FALSE] [DON'T KNOW]
4. Lower Respiratory Tract Infections from RSV is deprived of specific signs/symptoms	[TRUE] [FALSE] [DON'T KNOW]
5. In Europe, RSV season spans from …	
November – March	[ ]
October – February	[ ]
September – January	[ ]
6. SARS-CoV-2 and RSV have the same means of transmission	[TRUE] [FALSE] [DON'T KNOW]
7. Safe and effective vaccines against RSV are commercially available	[TRUE] [FALSE] [DON'T KNOW]
8. Monoclonal antibodies can be used against RSV only as immunoprophylaxis	[TRUE] [FALSE] [DON'T KNOW]
9. Immunoprophylaxis for RSV should be delivered …	
… every two months, during RSV season	[ ]
… every month, during RSV season	[ ]
… only at the beginning of RSV season	[ ]
10. Globally, RSV causes a total … deaths in children < 1 age	
43,800	[ ]
430,800	[ ]
Around 1,000,000	[ ]
11. According to available figures, RSV causes every year a total of … hospitalizations	
2 million	[ ]
10 million	[ ]
22 million	[ ]
Don't know	[ ]
12. According to WHO estimated, RSV causes … of Lower Respiratory Tract Infections	
… 40% …	[ ]
… 60% …	[ ]
… 75% …	[ ]
13. RSV infections may cause severe neurological complications	[TRUE] [FALSE] [DON'T KNOW]
14. RSV has been acknowledged as a risk factor for adult asthma	[TRUE] [FALSE] [DON'T KNOW]
15. Seroprevalence for RSV reaches 100% before 2nd year of age	[TRUE] [FALSE] [DON'T KNOW]
16. Maternal antibodies reduce the risk of RSV infections during first 120 days of life	[TRUE] [FALSE] [DON'T KNOW]
17. Hospitalization rate for RSV during the first year of age may reach…	
0.5 per 100	[ ]
1 per 100	[ ]
5 per 100	[ ]
18. The majority of patients hospitalized for RSV are affected by chronic respiratory disorders and cardiac malformations	[TRUE] [FALSE] [DON'T KNOW]
19. The majority of hospitalizations for RSV occur among pre-term infants	[TRUE] [FALSE] [DON'T KNOW]
20. According to available recommendations, mAb should be used only in preterm infants	[TRUE] [FALSE] [DON'T KNOW]
21. Around ¾ of all RSV-related deaths occurs in subjects older than 65 year	[TRUE] [FALSE] [DON'T KNOW]
22. During SARS-CoV-2 pandemic, global incidence of RSV infections has decreased	[TRUE] [FALSE] [DON'T KNOW]

23. During winter season 2021 has been affected by a RSV epidemic	[TRUE] [FALSE] [DON'T KNOW]
24. RSV natural infection elicit a long-lasting immunity	[TRUE] [FALSE] [DON'T KNOW]
25. Severe complications are more likely in RSV than in Seasonal Influenza infections	[TRUE] [FALSE] [DON'T KNOW]
3. Please rate the following items from "not significant" (1) to "very significant" (5)	
Ho do you perceive the frequency of RSV infections?	
… in infants [age 0 to 8 years]	[1] [2] [3] [4] [5]
… in adults [age 18 to 64 years]	[1] [2] [3] [4] [5]
… in elderly [age ≥ 65 years]	[1] [2] [3] [4] [5]
Ho do you perceive the severity of RSV infections?	
… in infants [age 0 to 8 years]	[1] [2] [3] [4] [5]
… in adults [age 18 to 64 years]	[1] [2] [3] [4] [5]
… in elderly [age ≥ 65 years]	[1] [2] [3] [4] [5]
4. Are you favorable towards the implementation of a RSV vaccine in the specific vaccine schedule, if commercially available? (1 = totally disagree; 5 = totally agree)	[1] [2] [3] [4] [5]
5. Are you favorable towards the implementation of a mAb prophylactic therapy against RSV (1 = totally disagree; 5 = totally agree)	[1] [2] [3] [4] [5]
6. In the use of mAb against RSV, which aspects are of specific importance, from your point of view? (1 = totally disagree; 5 = totally agree)	
… avoiding natural infections	[1] [2] [3] [4] [5]
… avoiding complications (i.e. LRTI)	[1] [2] [3] [4] [5]
7. Please provide some general information about you	
Year of birth:	_______________
Year of medical qualification:	_______________
You identify yourself as:	[Male][Female][No Answer]
Do you work in hospital settings?	[yes] [no] [no answer]

Notes: the present questionnaire can be shared and modified by the end user. Please cite the present paper.

Table S2. Knowledge test: summary of the answers on presented items proposed to the 389 Italian Pediatricians who participated into the present survey on Respiratory Syncytial Virus (RSV) (Cronbach's alpha = 0.701).

Statement	Correct Answer	Total (No./389)
Nearly all RSV infections occur in infants and children	FALSE	247, 63.5%
In most cases, infants acquire RSV infections from their parents	FALSE	196, 50.1%
In most cases, RSV evolves in an uncomplicated influenza-like illness	TRUE	329, 84.6%
Lower Respiratory Tract Infections from RSV is deprived of specific signs/symptoms	TRUE	202, 51.9%
In Europe, RSV season spans from …		
November – March	TRUE	239, 61.4%
October – February	FALSE	90, 23.1%
September – January	FALSE	46, 11.8%
Don't know	-	14, 3.6%
SARS-CoV-2 and RSV have the same means of transmission	TRUE	374, 96.1%
Safe and effective vaccines against RSV are commercially available	FALSE	306, 78.7%
Monoclonal antibodies can be used against RSV only as immunoprophylaxis	TRUE	170, 43.7%
Immunoprophylaxis for RSV should be delivered …		
… every two months, during RSV season	FALSE	60, 15.4%
… every month, during RSV season	TRUE	150, 38.6%
… only at the beginning of RSV season	FALSE	133, 34.2%
Don't know	-	46, 11.8%
Globally, RSV causes a total … deaths in children < 1 age		
43,800	TRUE	198, 50.9%
430,800	FALSE	127, 32.6%
Around 1,000,000	FALSE	25, 6.4%
Don't know	-	39, 10.0%
According to available figures, RSV causes every year a total of … hospitalizations		
2 million	TRUE	156, 40.1%
10 million	FALSE	157, 40.4%
22 million	FALSE	46, 11.8%
Don't know	-	30, 7.7%
According to WHO estimated, RSV causes … of Lower Respiratory Tract Infections		
… 40% …	FALSE	180, 46.3%
… 60% …	TRUE	164, 42.2%
… 75% …	FALSE	39, 10.0%
Don't know	-	6, 1.5%
RSV infections may cause severe neurological complications	TRUE	274, 70.4%
RSV has been acknowledged as a risk factor for adult asthma	TRUE	309, 79.4%
Seroprevalence for RSV reaches 100% before 2nd year of age	TRUE	230, 59.1%
Maternal antibodies reduce the risk of RSV infections during first 120 days of life	TRUE	299, 76.9%
Hospitalization rate for RSV during the first year of age may reach…		
0.5 per 100	TRUE	65, 16.7%
1 per 100	FALSE	102, 26.2%
5 per 100	FALSE	203, 52.2%
Don't know	-	19, 4.9%

The majority of patients hospitalized for RSV are affected by chronic respiratory disorders and cardiac malformations	FALSE	156, 40.1%
The majority of hospitalizations for RSV occur among pre-term infants	FALSE	99, 25.4%
According to available recommendations, mAb should be used only in preterm infants	TRUE	116, 29.8%
Around ¾ of all RSV-related deaths occurs in subjects older than 65 years	TRUE	87, 22.4%
During SARS-CoV-2 pandemic, global incidence of RSV infections has decreased	TRUE	252, 64.8%
During Winter Season 2021, Italy has been affected by a RSV epidemic	TRUE	285, 73.3%
RSV natural infection elicit a long-lasting immunity	FALSE	207, 53.2%
Severe complications are more likely in RSV than in Seasonal Influenza infections	TRUE	284, 73.0%

Table S3. Characteristics of the 389 Italian Pediatricians (PDL) participating into the present survey on knowledge, attitudes and practices on respiratory syncytial virus (RSV) by working or not in hospital settings.

**Variable**	**Working in Hospital Settings**		***p* value**
	Yes (No./106, %)	No (No./283, %)	
Age ≥ 40 years	18, 17.0%	152, 53.7%	< 0.001
Seniority ≥ 10 years	29, 27.4%	217, 76.7%	< 0.001
Male Gender	38, 35.8%	103, 36.4%	0.920
Region of residence			< 0.001
North-Western Italy	30, 28.3%	39, 13.8%	
North-Eastern Italy	28, 26.4%	99, 35.0%	
Central Italy	30, 28.3%	92, 32.5%	
Southern Italy	0, -	35, 12.4%	
Major Islands	18, 17.0%	18, 6.4%	
Higher Knowledge Status	52, 49.1%	127, 44.9%	0.461
Higher Risk Perception			
Infants	20, 18.9%	122, 43.1%	< 0.001
Adults	17, 16.0%	102, 36.0%	< 0.001
Elderly	37, 34.9%	150, 53.0%	0.001
Previously managed RSV cases	79, 74.5%	84, 29.7%	< 0.001
Previously delivered mAb	36, 34.0%	20, 7.1%	< 0.001
Favorable attitude towards mAb	73, 68.9%	218, 77.0%	0.099
Favorable towards implementation of RSV vaccine (when available)	106, 100%	255, 90.1%	0.001

Table S4. Correlation of relative search volumes (Google TrendsTM) on Respiratory Syncytial Virus (RSV) with General Knowledge Score (GKS) and Risk Perception Scores (RPS) (Spearman's correlation coefficient).

Variable	GKS	RPS infants	RPS adults	RPS elderly
Relative Search Volumes on RSV	0.025 ($p = 0.618$)	-0.052 ($p = 0.307$)	0.034 ($p = 0.500$)	0.025 ($p = 0.621$)
